# Implementation of Out-of-Office Blood Pressure Monitoring in the Netherlands: From Clinical Guidelines to Patients’ Adoption of Innovation

**DOI:** 10.1097/MD.0000000000001813

**Published:** 2015-10-30

**Authors:** Pricivel M. Carrera, Mattijs S. Lambooij

**Affiliations:** From the National Institute for Public Health and the Environment (RIVM), Department Quality of Care and Health Economics, Center for Nutrition, Prevention and Health Services, Bilthoven, The Netherlands.

## Abstract

Out-of-office blood pressure monitoring is promoted by various clinical guidelines toward properly diagnosing and effectively managing hypertension and engaging the patient in their care process. In the Netherlands, however, the Dutch cardiovascular risk management (CVRM) guidelines do not explicitly prescribe 24-hour ambulatory blood pressure measurement (ABPM) and home BP measurement (HBPM). The aim of this descriptive study was to develop an understanding of patients’ and physicians’ acceptance and use of out-of-office BP monitoring in the Netherlands given the CVRM recommendations.

Three small focus group discussions (FGDs) with patients and 1 FGD with physicians were conducted to explore the mechanisms behind the acceptance and use of out-of-office BP monitoring and reveal real-world challenges that limit the implementation of out-of-office BP monitoring methods. To facilitate the FGDs, an analytical framework based on the technology acceptance model (TAM), the theory of planned behavior and the model of personal computing utilization was developed to guide the FGDs and analysis of the transcriptions of each FGD.

ABPM was the out-of-office BP monitoring method prescribed by physicians and used by patients. HBPM was not offered to patients even with patients’ feedback of poor tolerance of ABPM. Even as there was little awareness about HBPM among patients, there were a few patients who owned and used sphygmomanometers. Patients professed and seemed to exhibit self-efficacy, whereas physicians had reservations about (all of their) patients’ self-efficacy in properly using ABPM. Since negative experience with ABPM impacted patients’ acceptance of ABPM, the interaction of factors that determined acceptance and use was found to be dynamic among patients but not for physicians.

In reference to the CVRM guidelines, physicians implemented out-of-office BP monitoring but showed a strong preference for ABPM even where there is poor tolerance of the method. We found that physicians’ positive attitude to ABPM enabled the use of the method by patients which, in turn, impeded the diffusion of HBPM. For patients, the acceptance process of HBPM can only begin after the physician has adopted the innovation. Physicians are in a position to encourage as well as hinder out-of-office BP monitoring and self-management.

## BACKGROUND

Self-management, including self-monitoring, with the support of patients’ healthcare providers, is emphasized in the management and control of many chronic conditions and their risk factors, notably cardiovascular disease (CVD) and high blood pressure (BP) or hypertension.^1–3^ The early and accurate diagnosis and management of hypertension with the engagement of the patient in their care process are expected, and have been shown to improve quality of care, deliver desired health outcomes, and moderate increases in health spending.^4,5^ Consequently, and in recognition of the limitations of office BP measurement (OBPM), various international clinical practice guidelines advocate out-of-office BP monitoring to improve the diagnosis and management of hypertension.^6–11^

Although convenient for the health professional, OBPM over-diagnoses an estimated 20% to 30% of cases and misses about one-third of those with masked uncontrolled hypertension, which presents as elevated BP outside of the clinic but normal BP in the clinic.^12^ In collecting automated BP readings over a 24-hour period, ambulatory BP measurement (ABPM) is particularly useful for and recommended in diagnosing hypertension especially in the case of white-coat or masked uncontrolled hypertension and night-time BP and dipping.^13^ Where patients have poor tolerance of ABPM, due to arm discomfort and sleep disturbance, and where treatment adherence and antihypertensive drug effects are of particular concern, home BP measurement (HBPM), which provides BP measurements over a period of 1 week, is recommended.^6^Table 1 compares and contrasts OBPM with out-of-office BP monitoring methods, ABPM and HBPM.

**TABLE 1:**
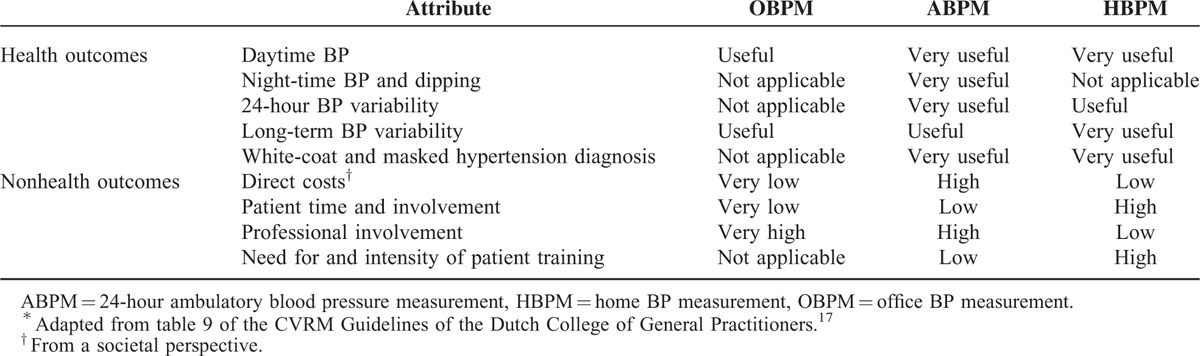
Comparison of OBPM, ABPM, and HBPM^∗^

Although it has been suggested that HBPM will increasingly replace ABPM in the diagnosis and management of hypertension given the advantages of HBPM over ABPM in terms of cost, availability and application,^14^ a systematic review with meta-analysis on the relative effectiveness of OBPM and HBPM compared with ABPM did not support either OBPM or HBPM as a single diagnostic test.^15^ The guidelines of the National Institute for Health and Clinical Excellence in England, the Japanese Society of Hypertension (JSH), National Heart Foundation of Australia (NHF) and the European Society of Hypertension advocate the complementary use of HBPM and ABPM.^6–11^ In fact, the JSH guidelines emphasize HBPM readings over OBPM readings, which appear to contribute to the widespread use of HBPM in Japan by physicians and patients alike.^11^ In comparison, the Dutch cardiovascular risk management (CVRM) guidelines do not explicitly prescribe HBPM or ABPM. The summary of the CVRM guidelines, actually, states that the measurement of BP outside of the clinic is optional.^16^ At the same time, however, the CVRM guidelines notes section discusses the advantages of HBPM over ABPM (and OBPM) and “recommends the use of ABPM only in specific circumstances”^17^ (p.21).

**FIGURE 1:**
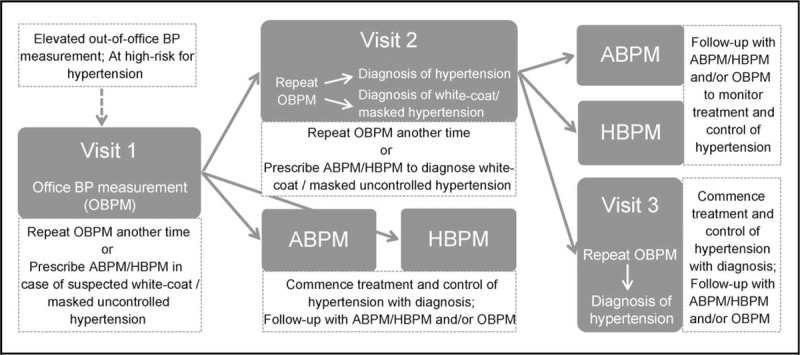
Illustrative use of OBPM, ABPM and HBPM according to the Cardiovascular Risk Management Guidelines.

Since the CVRM guidelines neither clearly enforce ABPM or HBPM nor discourage the use of OBPM, the practice of BP measurement and uptake of out-of-office hypertension monitoring may well be varied as shown in Figure 1. Figure 1 depicts the use of ABPM and HBPM in clinical practice in conjunction with OBPM and the choice of ABPM and/or HBPM in out-of-office BP monitoring. Since the CVRM guidelines are not explicit on the prescription of out-of-office BP, notwithstanding the assessment presented of OBPM, ABPM, and HBPM based on available evidence, the onset and degree of use of ABPM and/or HBPM is likely to be discrepant with potential adverse effect on the uptake of out-of-office hypertension monitoring and selfmonitoring in the Netherlands. Given the context, to know about the adoption of patients of out-of-office hypertension monitoring we need to know not just their acceptance and use but those of (their) physicians as well.

ABPM = 24-hour ambulatory blood pressure monitoring, HBPM = home BP monitoring, OBPM = office BP monitoring.

Previous studies have shown that while both physicians and patients positively regard ABPM and HBPM,^18,19^ interviews with health professionals, who took part in a randomized controlled trial on self-management of hypertension in England, found a lack of support for the wider uptake of HBPM among physicians (in combination with telemonitoring of BP measurements) as an innovation and of self-management in general.^18^ Such clinical inertia, of course, can but need not hinder out-of-office BP monitoring. Focus group discussions (FGDs) with care providers in Australia on strategies to improve the management of hypertension found that providers concerned about the validity of clinic BP readings started prescribing ABPM or HBPM.^20^ Although awareness of the NHF guidelines was found to be important in the implementation of out-of-office BP measurement, awareness was claimed to be only one of several factors that influenced providers’ acceptance and prescription of out-of-office BP monitoring methods.

A study of Dutch physicians’ adherence to clinical guidelines found a broad range of barriers and identified barriers related to attitude, behavior, and knowledge regarded as key barriers to the implementation of recommendations.^21^ Although the study covered the CVRM guidelines, unfortunately, it did not look into the method for measuring BP as a key recommendation. Given the Dutch context—of having guidelines that do not explicitly advocate the use of HBPM and ABPM, what factors determine patients’ and physicians’ acceptance and use of ABPM and HBPM? This study aimed to answer the following research questions:

1. What factors influenced the acceptance of out-of-office hypertension monitoring among patients and physicians in the Netherlands?
2. Which factors played a role in the actual use of out-of-office hypertension monitoring methods by patients vis-à-vis the prescription of these by physicians?
3. How did the use of out-of-office hypertension monitoring methods relate to clinical practice guidelines?

### Analytical Framework

To understand patients’ and physicians’ acceptance and use of ABPM and HBPM, an analytical framework was developed based on the technology acceptance model (TAM) by Davis^22^ and the theory of planned behavior (TPB) of Ajzen^23^ as well as the model of personal computing utilization by Thompson et al.^24^ The framework served as a practical tool to guide the development of the instrument that was used in the collection of qualitative data and their analysis by the researchers involved in the conduct of this study, namely H.F.—a postgraduate social science student with prior postgraduate degree in behavioral science and experience in health services research; PC—an assistant professor specializing in comparative health policy and health services research and M.L. a senior researcher and expert in implementation and health services research. Table 2 gives an overview of the analytical framework of the study and a sample of questions that were posed during the FGDs that were conducted (and discussed in the next section).

**TABLE 2:**
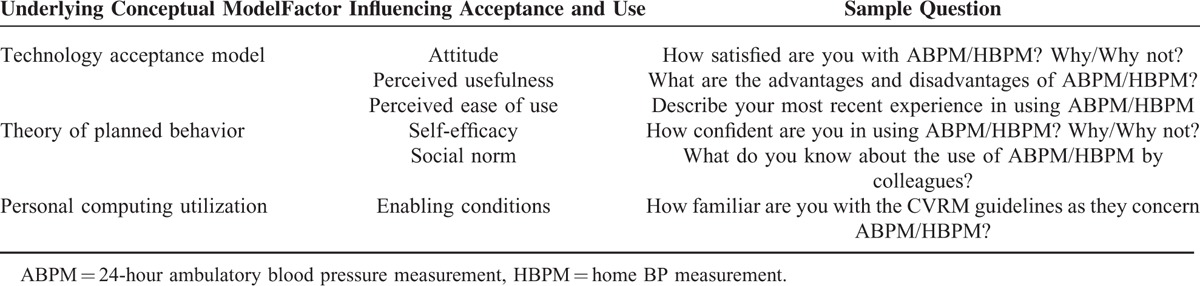
Analytical Framework as Basis for Development of Interview Guide

The TAM posits that the key to increasing the use of information technology (IT) is to first increase acceptance of IT, or technological innovation, in general. To increase acceptance, the factors that determine such need to be understood. An important factor, according to TAM is attitude, which is the sum of (positive and negative) beliefs weighted by evaluations of these beliefs. These beliefs are influenced by perceived usefulness, which is the degree to which an individual believes that usage would be beneficial, and perceived ease of use, or the degree to which an individual is convinced that usage would not be arduous. One's perception of the ease or difficulty of performing (or one's perceived behavioral control), is influenced by self-efficacy, which is the conviction that one can successfully effectuate outcomes.^25^ Attitude is likewise influenced by social norm, or the impact of one's social environment, and enabling conditions, which are objective factors in the environment that promote action.

Enabling conditions, as a factor, originates from the model of personal computing utilization by Thompson et al.^24^ In the context of this study, enabling conditions include the existence of pertinent clinical guidelines (ie, the CVRM guidelines) as well as the availability of devices used for BP monitoring and their affordability. Social norm, meanwhile, covers the prescription of out-of-office BP monitoring among physicians as a professional group as well as the use of either ABPM or HBPM by hypertensive family members, colleagues, and community members among patients. The acceptance of ABPM or HBPM leads to the prescription by the physician and the use of the method for out-of-office BP measurement by the patient, respectively.

### Methodology

FGDs were carried out in this descriptive study on patients’ and physicians’ acceptance of and experience with out-of-office BP monitoring. The FGD is a qualitative research method for eliciting information from specific population subgroups whose points-of-view may differ.^26^ The protocol for the study and the questionnaire used to facilitate the FGDs were submitted to the Medical Ethics Committee Twente for adjudication, which exempted the study from review as the nature of the study was found beyond the remit of the Medical Sciences Research with Human Subjects Act (Wet medisch-wetenschappelijk onderzoek met mensen) and that measures pertaining to confidentiality and informed consent were found appropriate.

While not meant to be representative, focus group research is particularly useful for understanding the mechanisms that come into play during implementation of diagnostic and therapeutic approaches in usual clinical practice settings.^27^ Since the purpose of the study is to gain an understanding of Dutch patients’ and physicians’ perspective and experiences and given the challenge of recruiting within these particular groups, small FGDs involving 5 to 6 participants, instead of the traditionally recommended size of 5 to 8, were arranged.^28^

Physician participants were contacted through an association of primary care physicians (ie, general practitioners, hereafter GPs) in the province of Overijssel in the eastern part of the Netherlands and recruited by phone. Hypertensive patients with experience in out-of-office BP monitoring were recruited through their GP as well as by social media to allow for a more diverse sample of respondents in terms of age (since hypertension is associated with advance age and homogeneity in age-groups among patient participants may result in identical perspectives and experiences). Recruitment continued until the FGDs generated very little new, relevant information for analysis. As elaborated in the Results Section, we also sought to recruit patients and physicians with experience with HBPM but we were unable to arrange any focus group on the use of HBPM during the 8 months of recruitment (from January to August 2014). An additional focus group with physicians was hampered by scheduling difficulties, and time constraints.

All FGDs were carried out by HF and PC and were audio-taped; HF facilitated each of the FGD sessions while PC observed the discussions and took notes. The first 2 FGDs served to validate the usefulness of the instrument developed and any modification necessary (based on postinterview discussions elaborated further in the next section). All FGD participants signed an informed consent form and were informed about the objectives and nature of the research and limitations concerning confidentiality. In particular, participants were informed that they could, at any point during the FGD, decide to withdraw but that it would not be possible to expunge statements they had (already) made during the course of the FGD before the start of the session. In addition, the active contribution of every participant was encouraged and emphasized at the commencement of every session to facilitate the contribution of all participants. Table 3 gives an overview of the details of the 4 FGDs conducted.

**TABLE 3:**
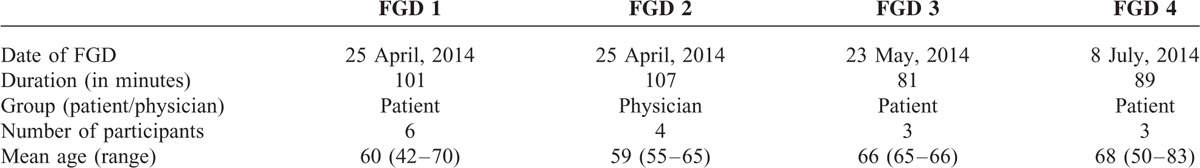
Description of Focus Group Discussion (FGD) Sessions

### Data Analysis

Postinterview discussions between HF and PC took place immediately after or soon after each FGD session to identify key findings as well as new relevant information that may be useful for the following focus group. Thereafter, PC and ML discussed key findings and their implications for the study. The audio-recordings of the FGD sessions were transcribed verbatim by a third-party transcribing company. The transcripts were then analyzed by qualitative content analysis with a directed approach.^29^ Directed content analysis aims to validate or conceptually extend a theoretical framework.^30^ The theoretical framework served as basis for the analysis of the data. Statements were classified by PC according to the factors identified as influencing acceptance and use. Findings were organized in a conceptually clustered matrix per FGD session, which allowed for comparisons between respondents and between sessions. The interpretation of results was carried out by both PC and ML. Disagreements were resolved by revisiting the transcripts and the source statements that served as the basis for interpretation.

## RESULTS

A key finding of this study is that no patient was prescribed, had heard of or experienced HBPM as a method for out-of-office BP monitoring although there were a few patients who had their own digital sphygmomanometers and used them. Patients came to know about ABPM through their GPs as no one in their respective social circles had used the method. Moreover, no patient stated that his/her GP offered HBPM as an option for BP monitoring outside of the clinic. Consequently, the evaluation of factors that influenced the acceptance and use of out-of-clinic BP monitoring focused on ABPM. Illustrative quotes from patients and physicians are presented in Table 4 and elaborated on below.

**TABLE 4:**
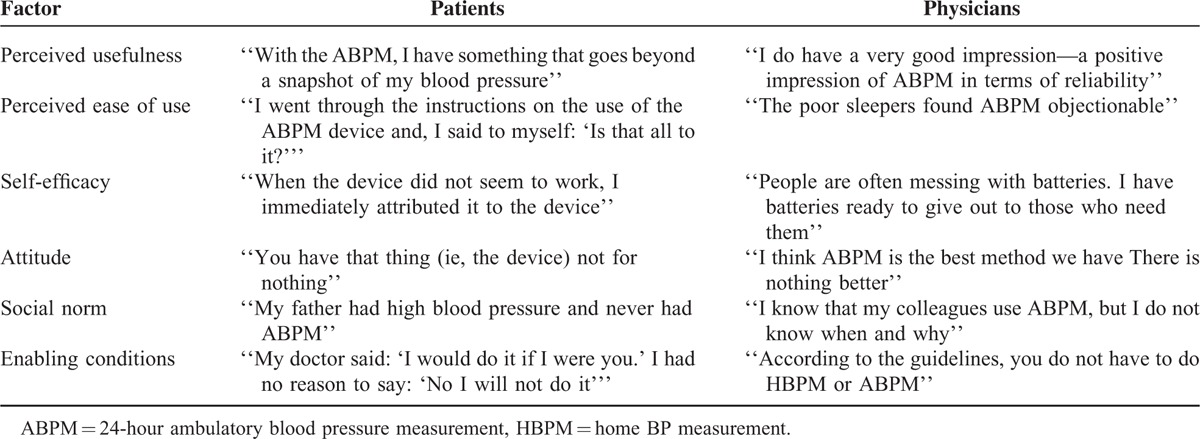
Illustrative Quotes From Patients and Physicians

### Attitude Toward ABPM and HBPM

Patients expressed an overall positive attitude toward ABPM—before and after use. Even though some patients did not tolerate ABPM well, patients’ attitude remained positive given the perceived usefulness of ABPM. They found HBPM interesting after being informed about the method in the course of the FGD. Patients felt that, in addition to being useful, HBPM would be easier to use and more effective since it would not intrude on their daily activities or cause pain and bruising. Patients who (already) had their own digital sphygmomanometer stated that they plan to carry out HBPM on their own volition as a complement to ABPM. Physicians, meanwhile, expressed very positive attitude toward ABPM even as they recognized the problems some patients had in using ABPM (discussed further below). They felt that HBPM was overall inferior to ABPM and not just in terms of nighttime BP and dipping.

### Perceived Usefulness of ABPM

Although the choice of out-of-office BP monitoring method was made by the health professional, the prescription of the method by the GP was seen as beneficial and valued by patients. Being prescribed ABPM conjured feelings of relief for some patients with the reduction or elimination of uncertainty about their condition and/or treatment while it conjured anxiety about what the prescription of ABPM implied for others. Physicians’ view of the virtues of ABPM led them to prescribe the method to their patients which, in turn, exerted a huge influence on patients’ acceptance and use of ABPM.

### Perceived Ease of Use of ABPM Device

While patients had an overall positive attitude toward ABPM, their experience in using the ABPM device was varied. As noted above, there were patients who experienced difficulties, which affected their attitude toward the method afterwards (ie, resulted to a less positive attitude). These difficulties pertained to the intrusion of the method on patients’ daily activities both at work and at home as well as physical discomfort. There were, at the same time, patients who stated that they had no problems in using the ABPM device. While patients initially saw the use of ABPM as straight forward, some said that it proved to be inconvenient to use. These practical inconveniences in using and adverse effects of ABPM use were relayed by them to their physicians.

### Patients’ Self-Efficacy and Physicians’ Perception Thereof

Ownership and use of sphygmomanometers (by a few patients) were associated with a more positive attitude toward ABPM. While patients said that they were fully able to self-measure and self-monitor (ie, they professed self-efficacy), which aided and reinforced the idea of ease of use of the method, physicians felt that not all of their patients were able to properly use ABPM and, thus, contribute effectively to self-measurement and self-monitoring. Physicians had their reservations about the self-efficacy of patients and their ability to do out-of-office BP measurements. According to them, patients are varied in their ability to manage even the use of the ABPM device.

### Social Norm

Physicians indicated a very positive attitude toward ABPM specifically, in terms of the reliability the method and the clinical utility of readings it generated, which they thought was shared by their immediate peers (ie, colleagues in the same shared practice or from continuing medical education courses). Unfortunately, they were not aware of the circumstances under which ABPM was prescribed and how their prescription of the method compared within the wider physician community. In comparison to physicians, patients did not know of anyone, family or friends, who had previously experienced or are currently using ABPM.

### The CVRM Guidelines as an Enabler

Physicians expressed reservations about the value of the guidelines in general. There appeared to be a selective uptake of the CVRM guidelines, given their acknowledgment of the benefits of ABPM over OBPM, but not in relation to HBPM. At the same time, however, there were indications of reliance on and adherence to the summary of the CVRM guidelines which favors OBPM. In addition to the guidelines, the reimbursement of the ABPM device—and not HBPM, was mentioned to have incentivized physicians’ prescription of ABPM for the use of their patients. Moreover, the cost of the device was noted as a major consideration in the choice of ABPM device.

## DISCUSSION

This study of the mechanisms behind patients’ and physicians’ acceptance and use of out-of-office BP monitoring in the Netherlands indicated that positive and strong acceptance and use of ABPM by patients and physicians alike may not be sufficient to facilitate wide scale diffusion of out-of-office BP monitoring. Even though our patients expressed intentions to increase their self-management of hypertension, there was little awareness among patients regarding HBPM since they were not told about the option by their GPs. Physicians did not seem to hold HBPM in as much regard as ABPM; neither did they prescribe HBPM even to patients for whom ABPM is not effective as recommended by the CVRM guidelines. Consequently, patients were prevented from determining whether, in their particular circumstance, HBPM is (more) effective and if they would consider the adoption of the method.

Table 5 summarizes the relevance and significance of factors that determine acceptance and use of out-of-office BP monitoring based on the analytical framework developed for this study. Attitude, perceived usefulness, perceived ease of use and enabling conditions were found to be major facilitators in the prescription by physicians of ABPM and its acceptance and use by patients. Whereas self-efficacy was a major factor on the part of patients, it only played a minor role on the part of physicians. Finally, while social norm was a factor for physicians’ prescription of ABPM, albeit minor, it did not facilitate the acceptance and use of ABPM among patients. The technological context appeared to drive acceptance and use for both patients and doctors; enabling conditions encouraged perceived usefulness and perceived ease of use, which then affected attitudes.

**TABLE 5:**
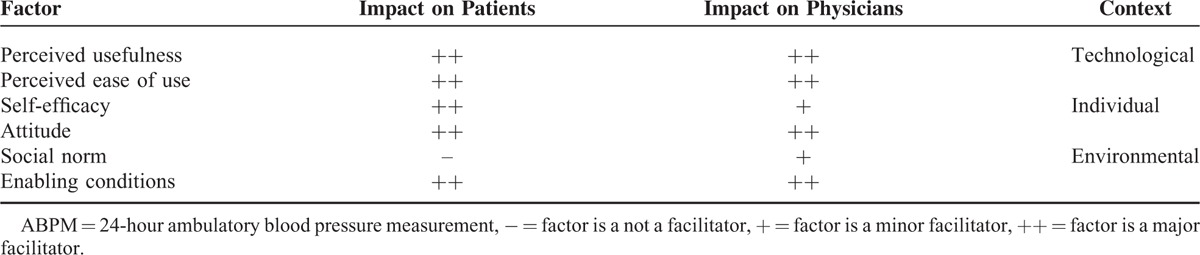
Determinants of Acceptance and Use of ABPM Among Patients and Physicians

In addition to differences in the significance of factors, the interaction of factors between patients and physicians also differed. The interaction of factors was not dynamic in the case of physicians—compared to patients—since physicians continued to prescribe ABPM. Whereas the CVRM guidelines advice the use of both ABPM and HBPM, and in fact relegated the use of ABPM in specific cases, we found that ABPM was the preferred method by GPs. This is in line with previous studies which found that physicians preferred OBPM or ABPM over HBPM even as they encouraged and supported self-measurement.^19,31^ In the Dutch context, however, physicians were consistent in advocating for their choice of BP monitoring method with their patients. To be sure, there is the danger, that continued prescription of ABPM by physicians to patients no longer accepting of the method might make them less adherent, thereby deteriorating the quality of the reading, the usefulness of the information and cost-effectiveness of hypertensive treatment.^32,33^

Our findings regarding the factors that affected acceptance of ABPM and HBPM are partly in line with previous findings that showed physicians’ local environment^34^ and preferences^35^ impact the adoption of innovation and that these factors interact.^20,36^ Our results also suggest that the skepticism of GPs about the self-efficacy of patients may have a bearing on their partiality to ABPM. This undermines patient empowerment which supports patient self-management by encouraging patients to codetermine decisions regarding their health and healthcare.^3,37^ Nonetheless, patients’ reliance on the physician for information is not absolute nor enduring, given the wider availability of such online and delivered through mobile communication and IT.^38^ It is worth reiterating that after having been told about HBPM self-monitoring, patients indicated their willingness and motivation to explore the method.

Given the huge influence of physicians in the choice of out-of-office BP measurement method, physicians are in a position to both encourage as well as hinder out-of-office BP monitoring and self-management. We found that physicians’ attitude enabled the use of ABPM by patients while it impeded the use of HBPM. This is an illustration of the importance of professional “buy-in” in the uptake of an innovation and change in practice.^39^ Two strategies may be used to convince physicians to use HBPM in their daily practice: focusing on the benefits of the innovation,^40^ or using peer feedback from successful use^41^ since “doctors typically learn in social contexts by doing and by interacting with peers”^36^ (pp.1388). These strategies, however, might require the affirmation of out-of-office BP monitoring in the CVRM guidelines since the guidelines neither clearly enforce ABPM or HBPM nor discourage the use of OBPM and the specificity of recommendations have been shown to be important in their implementation.^42^

Our FGDs did not include any physicians who prescribed HBPM as well as patients with experience in HBPM, despite our recruitment efforts. We initially sought to have an additional FGD session with physicians to validate our findings but failing to recruit patients with experience in HBPM and given scheduling difficulties and time constraints with additional physicians (who have prescribed use of ABPM), we took our findings from the FGDs to be suggestive of the nonacceptance of HBPM among GPs as not just limited to our sample. Since qualitative research is not quantitatively projectable to a larger population and given local area variation in practice, a study in another part of the Netherlands is advised to see whether ABPM is likewise preferred over HBPM and what drives the preferences of physicians. Given potential recruitment challenges, however, it might be worthwhile to employ other data collection methods than face-to-face focus group.^43^

Meanwhile, since we included participants who were willing to take part in the research, we may have selected GPs and patients who were relatively more positive about out-of-office hypertension monitoring. Since our goal is to reveal real-world challenges that limit the implementation of out-of-office BP monitoring, the acceptance and use of ABPM, which after 4 FGD sessions have indicated to suggest a point of saturation, allude to the complexity of implementation which our research has helped unravel. To validate our findings, and investigate their generalizability (among physicians and hypertensive patients in the Netherlands), a larger scale survey, ideally involving a discrete choice experiment using our analytical framework, is an important next step.^40,44^ It would be instructive to cover in such a study the impact of the ambiguity and ambivalence in the CVRM guidelines with respect to the use of methods for BP measurement on physicians choice of method as well as recommendations that unequivocally support out-of-office BP monitoring.

## CONCLUSION

This study sought to investigate patients’ and physicians’ use and acceptance of out-of-office BP monitoring in the Netherlands. For ABPM we were able to identify the factors that influenced acceptance by patients and physicians, namely: enabling conditions, perceived usefulness, perceived ease of use, and attitude. There were notable differences in the adoption processes of patients and physicians. Whereas self-efficacy was a major factor on the part of patients, it only played a minor role on the part of physicians. While social norm was a factor for physicians’ prescription of ABPM, it did not facilitate the acceptance and use of ABPM among patients. Social norm played a minor role, however. Even as the CVRM guidelines apply to both ABPM and HBPM, a markedly different acceptance of the 2 methods of out-of-clinic BP monitoring was noted.

The case presented in this study shows that if the added value of an innovation is perceived wanting or missing and incentives to change practice lacking,^45^ the adoption of the particular innovation may be stalled or hindered.^46^ Our study revealed a mechanism that might help us understand why the diffusion of HBPM as an innovation, and possibly other innovations in healthcare in the Netherlands, is slower than expected or different from the practice elsewhere. For patients, the acceptance process of HBPM appears to be contingent on their GP's adoption of the innovation. For GPs to adopt HBPM, ambiguity and ambivalence in the (use of methods as per) guidelines and lack of incentives to change seem to serve as important impediments.

Considering that both formal structural aspects such as clinical guidelines, and informal aspects regarding the attitude of physicians and patients have been found to affect the implementation of out-of-office BP monitoring, our findings underscore the complexity of implementing out-of-office BP monitoring, toward the self-management of patients within and between health systems. Granted that adherence to clinical practice guidelines is an issue on its own, to be effective in advancing out-of-office BP monitoring, recommendations on BP monitoring need to be coherent and clear. Otherwise, the uptake of out-of-office BP monitoring may be inconsistent and suboptimal. At the same time, since guidelines alone are unlikely to result to the diffusion of innovation, other determinants pertaining to end users need to be addressed. Why are physicians and patients not adopting an innovation and how can we make them do so are questions we need to ask if we want continuous improvement in and patient-centered healthcare.
